# Family members' perceptions of counselling during visits to loved ones in an adult ICU

**DOI:** 10.1002/nop2.1738

**Published:** 2023-04-05

**Authors:** Minna Vanhanen, Merja H. Meriläinen, Tero Ala‐Kokko, Helvi Kyngäs, Pirjo Kaakinen

**Affiliations:** ^1^ Research Unit of Health Sciences and Technology University of Oulu Oulu Finland; ^2^ Oulu University of Applied Sciences Oulu Finland; ^3^ Wellbeing Services County of North Ostrobothnia, Medical Research Center Oulu Oulu University Hospital Oulu Finland; ^4^ Oulu University Hospital Oulu Finland; ^5^ Oulu University Hospital, Medical Research Center Oulu University Medical Faculty, Research Group of Intensive Care Medicine Oulu University Hospital University of Oulu and Medical Research Center (MRC) Oulu Finland

**Keywords:** adults ICU, counselling, family‐centred, intensive care

## Abstract

**Aims:**

The study's aims were to (1) assess family members' perceptions of the quality of the counselling they received while visiting a loved one in an adult ICU and (2) identify factors that influence family members' perceptions of counselling quality.

**Design:**

A cross‐sectional survey of visiting family members of adult ICU patients.

**Methods:**

Family members (*n* = 55) at eight ICUs across five Finnish university hospitals completed a cross‐sectional survey.

**Results:**

Family members assessed the quality of counselling in adult ICUs to be good. Factors associated with the quality of counselling were knowledge, family‐centred counselling, and interaction. Family members' ability to live normally was associated with understanding of the loved one's situation (*ρ* = 0.715, *p* < 0.001). Interaction was associated with understanding (*ρ* = 0.715, *p* < 0.001). Family members felt that intensive care professionals did not adequately ensure that they understood counselling‐related issues and that they lacked opportunities to give feedback, in 29% of cases, staff asked the family members whether they understood the counselling and 43% of family members had opportunities to offer feedback. However, the family members felt that the counselling they received during ICU visits was beneficial.

## 1 INTRODUCTION

Family members of adult intensive care unit (ICU) patients play both receptive and participatory roles when visiting the patient (Frivold et al., 2015), both in the patient's room and outside of it (DiSabatino & Custard, 2014; Erikson et al., 2011). These roles are enacted in an environment of unknowns (Minton et al., 2019) and give rise to physical and psychological experiences that may adversely affect the family members' daily lives, potentially leading to social isolation, relationship problems, and issues affecting employment and health such as anxiety, depression, and post‐traumatic stress disorder (Johansson, 2014; Kynoch et al., 2016, 2019; Lemiale et al., 2010; Linnarsson et al., 2010). Family members need knowledge about their loved one's diagnosis, treatment, prognosis, clinical condition, schedule of treatment, and goals, and knowledge about the treatment team and ways in which family members can participate in the caring process (Akdagli et al., 2020; Wilson et al., 2015). In addition, family members need counselling to calm and comfort them so they can gather their resources and engage in cultural and spiritual cooperation with intensive care professionals (ICPs) (de Beer & Brysiewicz, 2016). Family members' satisfaction with care increased if they felt that they had received adequate knowledge and support and had opportunities to participate in caring for their loved ones (Frivold et al., 2015). To improve the counselling offered to family members during ICU visits and ensure that their needs are met, it is essential to first assess their perceptions of the counselling that is currently received.

## 2 BACKGROUND

Family‐centred care is an approach to health care that is respectful of and responsive to individual family members' needs and values (Davidson et al., 2017). One of its requirements is that counselling for patients' family members should be planned and implemented in a family‐centred manner (Kääriäinen, 2007; Kääriäinen & Kyngäs, 2010; Kyngäs et al., 2017; Mattila et al., 2014; Rajala, 2018). Family members counselling is a broad term for the education, guidance, tips and information that family members receive from intensive care professionals (ICPs) to help them cope with their situation. In this study, ‘counselling’ is used specifically in reference to mutual interactions between family members and ICPs, while ‘information’ refers to materials and explanations given to family members by ICPs. The quality of the counselling that family members receive can be assessed in terms of its content, implementation, benefits, and allocated resources (Kääriäinen, 2007; Kaakinen et al., 2017; Kivelä et al., 2014).

Previous studies on this topic have yielded several valuable findings. Kynoch et al. (2016) concluded that family members' counselling needs can be divided into five categories: support, strengthening hope, proximity, need for counselling, and comfort. A later study by the same group on the experiences and needs of families with loved ones admitted to an ICU highlighted the importance of family‐centred interactions and strong relationships between ICPs and family members (Kynoch et al., 2021). Active interaction with ICPs during family members' counselling can benefit family members by increasing their comfort and strengthening their feeling of being actively involved in caring for their loved ones (Alfheim et al., 2019; Smithburger et al., 2017). The benefits of family members' counselling could potentially be enhanced by implementing goal‐oriented counselling (Curtis et al., 2011). Family members' positive attitudes to decision‐making were positively related to their ability to live a normal lifestyle, maintain hope, and interact comfortably with professionals (Iverson et al., 2014). Moreover, family members want to be involved in caring for their loved one (e.g., in delirium prevention) without obstructing or burdening ICPs (Smithburger et al., 2017). Finally, there is evidence that involving family members in acute care can promote better health outcomes and improve the satisfaction of both patients and family members (Clark et al., 2016; Cypress, 2011).

The need for counselling among family members of ICU patients has several causes. First, a lack of counselling has been linked to poor comprehension of the patient's diagnosis, prognosis, and treatment (Wetzig & Mitchell, 2017). Second, many family members experience a sense of unreality and confusion upon first seeing their loved ones in the hospital; receiving counselling on the loved one's condition can help them deal with this experience (McKiernan & McCarthy, 2010). Third, ICU patients are often unable to interact physically with family members as they normally would, so family members find it difficult to decide how to respond to and interact with their loved ones (Erikson et al., 2010). A problem is that family members' needs for counselling and assurance are not always met even when they are correctly identified by an ICP (Agard & Harder 2007; Kirchhoff et al., 2004; Scott et al., 2019; Verhaeghe et al., 2005), which may adversely affect their satisfaction with care and their mental health (Scott et al., 2019). For all these reasons, it is important to assess the quality of the counselling available to family members visiting loved ones in ICUs and find ways in which it can be improved to better meet their needs.

## 3 THE STUDY

### 3.1 Aim and objective

The aims of this study were to (i) assess family members' perceptions of the quality of counselling they received while visiting a loved one in an adult ICU, and (ii) identify factors that influence the quality of counselling for family members.

The following research questions were addressed:

1. How do family members assess the quality of counselling they received while visiting a loved one in an ICU?
2. Which factors do family members feel to be associated with the quality of counselling they received while visiting a loved one in an ICU?

## 4 METHODS

### 4.1 Design

The study used a quantitative cross‐sectional design. Its results are reported in accordance with the strengthening of the reporting of observational studies in epidemiology (STROBE) statement (Egger et al., 2008).

### 4.2 Method

Data were collected using the Counselling Quality Instrument for family members (CQIF) (© Kääriäinen, 2007), which was previously shown to have acceptable internal consistency and structural validity (Kääriäinen, 2007; Kaakinen et al., 2020; Rajala, 2018). The instrument includes seven background questions together with items relating to four sub‐dimensions: content of counselling (27 items), implementation of counselling (26 items), benefits of counselling (19 items), and resources for counselling (13 items). Respondents scored each item using a four‐point Likert scale (1 = totally satisfied, 4 = totally unsatisfied). An additional item scored on a five‐point Likert scale (1 = poor, 5 = excellent) asked respondents to assess the overall quality of the adult counselling they received in the ICU. The instrument developer modified the instrument's content to fit the study group. Its content validity was tested by seven experts in counselling, who assessed the questionnaire's items using a four‐point scale measuring relevance (1 = not relevant, 4 = highly relevant) (Polit & Beck, 2018). Content Validity Indices (CVIs) were then calculated for each section of the questionnaire based on the expert panel's responses and a CVI value >0.79 was taken as the threshold for acceptable content validity (Polit et al., 2007; Polit & Beck, 2006).

Questionnaires were posted to research coordinators at all eight adult ICUs belonging to university hospitals in Finland. Data were collected between September and December 2017. The research assistant submitted questionnaires and instructions to adult ICUs at university hospitals. Convenience sampling was used and nurses distributed the questionnaires to all family members within 3 days of their loved one's admission to the relevant unit. Respondents who completed the questionnaire placed it in a sealed envelope and deposited it in a locked box at the ICU for collection. The sole inclusion criterion was being a family member (i.e., a biological, legal, or emotional relative) of an ICU patient. Respondents were family members visiting adult intensive care units at eight University Hospitals in Finland. The first author maintained active contact with the research coordinators to increase the response rate. A total of 100 questionnaires were distributed to the university hospitals and the response rate was 55%.

### 4.3 Analysis

The quality of family members' counselling in intensive care was analysed in terms of its content, implementation, and benefits. For this purpose, 13 sum variables were developed based on the CQIF. The internal consistency of these sum variables was assessed by computing their Cronbach's alpha values, which ranged from 0.67 to 0.92 (Table 1).

**TABLE 1:** Sum variables and their Cronbach's alpha values.

Sum variables	Cronbach's alpha	Background variable	*p*
Content of counselling upon arrival at the ICU			
Knowledge of the loved one's current care	0.787		
Support for family member	0.827		
Content of bedside counselling at the ICU			
Knowledge of disease and the ICU environment	0.776		0.004^a^
Knowledge of recovery	0.812	Age	0.023^a^
Knowledge of medical aids and follow‐up care	0.722	Age	
Implementation of bedside counselling at the ICU			
Interaction during counselling	0.844		
Family‐centred counselling	0.809		
Atmosphere during counselling	0.649		
Benefits of counselling in the ICU			
Impact on the understanding of the loved one's situation	0.924	Arrival	0.023^b^
Impact on family members' attitudes to intensive care	0.861		
Ability to live as normal a life as possible	0.817		
Counselling resources			
ICPs' competence	0.751		
Ability to provide information in a timely manner	0.673		

a Mann–Whitney.

b Kruskal‐Wallis.

Data were analysed using IBM SPSS Statistics 25 (INM Corporation, Armonk, NY). Descriptive statistics (frequencies and percentages) were computed and missing values were replaced. Principal component analysis (PCA) with varimax rotation was used to analyse the relationships between the variables (Polit & Beck, 2018). Sum variables were created based on the results of a factor analysis.

The responses were grouped into two categories: scores of 1.00–1.99 and 2.00–4.00 represented satisfactory and unsatisfactory counselling, respectively. The significance of relationships between background and sum variables was assessed using the Mann–Whitney U and Kruskal–Wallis tests. Associations between non‐normally distributed sum variables relating to family members' perceptions of counselling quality were analysed using Spearman's rho and the coefficient of determination (*R* squared) to determine whether trends in one variable could be explained by trends in another. The threshold for statistical significance was set at *p* < 0.05 (Polit & Beck, 2018).

### 4.4 Ethics

The study was approved by the Research Ethics Committee and authorized by all five of Finland's university hospitals. The study design satisfied the privacy requirements of the General Data Protection Regulation (GDPR, 2018). Respondents were not pressured to participate in the study; their participation was entirely voluntary and anonymous. The researchers were in no way able to identify the respondents. Completed questionnaires were stored in a locked locker, no personal data were collected, and after conversion into digital format, the data were password‐protected on a predesignated computer accessible only to the researchers.

## 5 RESULTS

The family counselling received in the adult ICUs of Finnish university hospitals was assessed to be good, with a mean score of 4.61 on a scale ranging from 1 (poor) to 5 (excellent). The mean age of the respondents was 53 years (SD = 15; Table 2) and most of them were female (70%). Compared to respondents aged over 50 years, younger respondents were less satisfied with the knowledge they acquired through counselling about the loved one's disease, the ICU environment and the loved one's recovery (Table 1).

**TABLE 2:** Background information on participating family members (*n* = 55).

	*n*	%
Family members age		
20–39	11	20
40–59	21	38
60–79	22	40
How did patient reach the ICU?		
From home, via emergency room	29	52.7
From another ward of the same hospital	17	30.9
From a ward in a different hospital	9	16.4
Predictability of patient's ICU admission		
Loved one's need for intensive care was an unforeseen surprise	43	78.2
Loved one's need for intensive care was recognized as a possibility during hospitalization	6	10.9
Intensive care was elective and family members knew about it before the loved one's hospitalization	6	10.9
Duration of patient's ICU stay		
Days	26	47.3
Weeks	21	38.2
Months	3	5.5
Unknown to family members	5	9.1

### 5.1 Family members' perceptions and factors associated with the quality of counselling in adult intensive care units

#### 5.1.1 Content of counselling

Family members were generally satisfied with content of the counselling they received upon arrival at the ICU. The respondents reported 98% satisfaction with the knowledge they received about their loved one's current care before their bedside visit, 98% satisfaction with the knowledge acquired about equipment, aids and examinations used in their loved one's care, 100% satisfaction with the knowledge gained about limitations on their loved one's movement, and 90% satisfaction with the psychological support they received. Family members were also satisfied with the knowledge they gained about the duration of their loved one's ICU stay (91%) but were less satisfied with the knowledge received about the duration of the loved one's stay in the hospital after leaving the ICU (79%) and the care they would need after leaving the hospital (69%).

During bedside visits, family members were satisfied with the knowledge they received about pain management for loved ones (100%), examinations (98%), equipment (98%) and medical aids used in their loved one's care and follow‐up (98%). They were also satisfied with their opportunities to visit the loved one's bedside (96%) and the duration of the loved one's intensive care (91%). Family members were less satisfied with the knowledge they received about risk factors for recovery (80%), preventing infection (83%), accepting the loved one's changed appearance (86%), and knowledge about post‐ICU care (81%).

**FIGURE 1:**
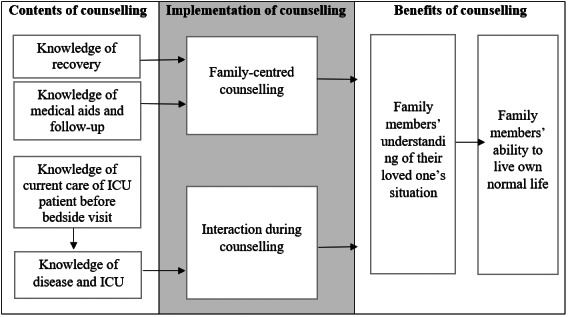
Understanding the loved one's situation was the main factor influencing family members' ability to live their own lives normally.

Knowledge about the loved one's current care before making a bedside visit was significantly associated with knowledge of the loved one's disease and the ICU environment (*ρ* = 0.747, *R* ^2^ = 0.56, *p* < 0.001) during the bedside visit. Both types of knowledge were significantly associated with the interaction during counselling (*ρ* = 0.596, *R* ^2^ = 0.36, *p* < 0.001; Figure 1).

#### 5.1.2 Implementation of family counselling

Half of the participating family members (56%) received family‐centred counselling. Table 3 shows the proportion of family members expressing satisfaction with different aspects of the implementation of counselling.

**TABLE 3:** Proportions of family members expressing satisfaction with different aspects of the implementation of counselling.

	%
Family‐centred counselling	
Content of counselling is adapted to the situation of the family members	56
Counselling provided emotional support and knowledge	100
Family members received adequate spiritual support	39
Interaction between families and ICPs	
ICPs asked whether family members understood issues discussed in counselling	29
Family members had opportunities to give staff feedback about counselling	43
Key issues were repeated at the end of family counselling	44
The language used during counselling was clear and understandable	90
Atmosphere during counselling	
The atmosphere during counselling was safe and open	90
ICPs had enough time for counselling	94
Family members had opportunities to ask questions	96

Family‐centred counselling was significantly associated with knowledge of recovery (*ρ* = 0.613, *R* ^2^ = 0.38, *p* < 0.001) and knowledge of medical aids and follow‐up care (*ρ* = 0.595, *p* < 0.001). Interaction during counselling was significantly associated with family‐centred counselling (*ρ* = 0.624, *R* ^2^ = 0.39, *p* < 0.001; Figure 1).

Interaction during counselling (*ρ* = 0.715, *R* ^2^ = 0.51, *p* < 0.001) and family‐centred counselling (*ρ* = 0.620, *R* ^2^ = 0.38, *p* < 0.001) were significantly associated with understanding the loved one's situation. Family members' ability to live a normal life was associated with understanding of the loved one's situation (*ρ* = 0.715, *R* ^2^ = 0,51, *p* < 0.001; Figure 1).

#### 5.1.3 Benefits of counselling for family members

Family members were generally satisfied with the benefits of counselling. They felt that counselling improved their understanding of the reasons for their loved one's admission to the ICU (91%) and of intensive care in general (93%). In addition, most respondents felt that the counselling encouraged family members to meet their loved one in the unfamiliar ICU environment (86%), improved their understanding of intensive care (93%), and promoted a positive attitude towards ICPs (87%).

Counselling had stronger positive benefits on attitudes towards intensive care among respondents whose loved one had been admitted to the ICU from home (via the emergency room) than among those whose loved one was admitted electively or from another ward or hospital (Table 1).

#### 5.1.4 Resources for family counselling

Family members stated that their need for counselling was strongest when their loved one was first moved to the ICU for treatment (91%). They also agreed that they needed a summary of what had happened to their loved one before being moved to the ward. Half of the respondents (54%) felt that they received up‐to‐date counselling material.

## 6 DISCUSSION

Family members considered the quality of counselling in the studied ICUs to be good. Factors related to the quality of counselling were knowledge, family‐centred counselling, and interaction. The family members felt that the counselling they received during ICU visits was beneficial. Respondents were also satisfied with the content and benefits of the counselling they received while visiting their loved one in an adult ICU. They were most dissatisfied with the implementation of counselling and the availability of up‐to‐date counselling material. The counselling of family members in an adult ICU is an integrated process that can be divided into pre‐visit counselling (e.g. in the family room) and bedside counselling. It is important to assess satisfaction with counselling in both phases because family members of adult ICU patients play both receptive and participatory roles (Frivold et al., 2015) inside and outside the patient's room (DiSabatino & Custard, 2014; Erikson et al., 2011). The results obtained in this study show that family members were generally satisfied with the knowledge of the loved one's care that they received at the ICU both before and during their bedside visits. Family members considered learning about the ICU patient's care to be the most important aspect of pre‐visit counselling because it allowed them to better engage with subsequent counselling on disease, the ICU environment, and recovery during the visit.

Interaction during counselling was associated with the family members' satisfaction with two aspects of counselling content: knowledge of the loved one's current care before making a bedside visit and knowledge of disease and the ICU environment during the bedside visit. Previous studies have found that family members of ICU patients often feel that they have a poor understanding of the patient's diagnosis, prognosis, and treatment, which may be explained by a lack of counselling from ICPs or unclear counselling (Wetzig & Mitchell, 2017). Our results indicate that the content of family members' counselling becomes more complex as the family members go from their initial arrival at the ICU to the bedside visit with the patient. According to previous studies, counselling should give family members knowledge about their loved one's diagnosis, treatment, prognosis and clinical condition, and treatment schedules and goals, the treatment team, and how family members can participate in care (Akdagli et al., 2020; Wilson et al., 2015). Counselling on such diverse topics is important and beneficial because family members' satisfaction with care increases when they believe they have received detailed knowledge and support and have opportunities to participate in caring for their loved one (Frivold et al., 2015).

Family‐centred care is an approach to health care that is respectful of and responsive to individual family members' needs and values (Davidson et al., 2017). It requires counselling given to patients' family members to be implemented in a family‐centred manner (Kääriäinen, 2007; Kääriäinen & Kyngäs, 2010; Kyngäs et al., 2017; Mattila et al., 2014; Rajala, 2018). The results presented here indicate that family‐centred counselling that provides knowledge of recovery and follow‐up allows family members to develop their understanding of their individual situation and that of their loved ones. The aspect of counselling quality with which family members were least satisfied was the implementation of counselling; family members felt that ICPs did not take sufficient care to verify that they understood the issues discussed during counselling and that they lacked opportunities to offer feedback. However, they did feel able to ask questions during counselling in a safe and open atmosphere (provided that they knew what to ask). Previous studies have found that family members often feel that ICPs assess their needs incorrectly (Agard & Harder, 2007; Kirchhoff et al., 2004; Scott et al., 2019; Verhaeghe et al., 2005). The challenge for ICPs is to ensure that they adopt a holistic approach to the content of counselling, providing information on both coping in the present and recovery in the future. According to family members, it is not sufficient for counselling to involve only providing information; it should be a dialogue‐based process in which the transfer of knowledge is just one element. Family members also want to be involved in caring for their loved one (e.g., in delirium prevention) without obstructing or burdening ICPs (Smithburger et al., 2017). Therefore, open interaction with bedside ICPs can help family members by increasing their comfort and involvement in care (Alfheim et al., 2019; Smithburger et al., 2017).

Family‐centred counselling and interaction based on knowledge provided to family members were associated with understanding the loved one's situation. Understanding was in turn the main factor affecting the family members' ability to continue living their lives (e.g. by working), which was seen as the key measure of counselling quality. This is consistent with previous reports indicating that counselling on the patient's situation helps family members deal with the experience of having a loved one in intensive care (McKiernan & McCarthy, 2010). Previous studies have also shown that counselling is important for establishing good relationships between ICPs and family members (Kynoch et al., 2021) and promotes a positive attitude to ICU staff (Iverson et al., 2014). In addition, increasing family members' understanding of the reasons for their loved one's admission to the ICU and understanding of intensive care encourages them to engage with the loved one in the unfamiliar ICU environment. Although ICU patients' stays in the ICU are generally quite short (only 47% spend more than 1 day in the ICU), it is important for ICPs to be prepared to offer counselling. The benefits of family members' counselling could potentially be enhanced if it was implemented in a goal‐oriented manner (Curtis et al., 2011).

Further research on interventions (e.g. training for ICPs) to strengthen dialogue‐based counselling is needed to identify effective counselling methods for use in adult ICUs. Most of the family members participating in our study were over 40 years old (78%) and had not anticipated their loved one's admission to the ICU (53%). Information on this issue could be provided to ICPs to help them when interacting with family members. There is a need to clarify the counselling needs of family members of different ages, particularly given that younger respondents reported lower satisfaction with counselling than older respondents in this study.

### 6.1 Limitations

The study has two notable limitations. First, the respondents were not randomly selected. Second, the number of respondents was somewhat low, which may limit the generalizability of the study results. Completed questionnaires were received from 55 family members with relatives being treated at ICUs in five university hospitals. The response rate was acceptable given that average survey response rates are typically between 20% and 40%. (Polit & Beck, 2018). Strengths of the study include the use of a questionnaire (QCI) which showed adequate content validity in this study, and which has shown good content validity in numerous previous studies (Kääriäinen, 2007; Kaakinen et al., 2017; Kivelä et al., 2014), and the Cronbach's alpha values ranged from 0.67 to 0.92 indicate good internal consistency. The questionnaire was modified for this study, after which its content validity was verified by expert assessment and by calculating its content validity index (S‐CVI). The S‐CVI was 0.89, which is above the threshold of acceptability (Polit & Beck, 2018). Only completed questionnaires were assessed during the analysis because responses to all items were needed to cover all areas of counselling; this is a notable weakness of the questionnaire used in this work. Therefore, although five incompletely filled‐out questionnaires were received, their data were excluded from the analysis.

## 7 CONCLUSION

The study confirms that the provision of high‐quality counselling during visits to loved ones in an adult ICU has beneficial effects according to family members. However, it also indicates that there is a need for improvement of implementation of counselling; family members felt that ICPs did not adequately ensure that they understood nurses the issues discussed during counselling and that they lacked the opportunity to provide feedback. Larger studies are needed to evaluate the effectiveness of family members' counselling in ICUs.

### 7.1 Clinical significance of the study

The family members' perceptions presented herein indicate that counselling has beneficial effects for adults visiting a loved one in intensive care. To improve the quality of the counselling provided to family members of ICU patients, ICPs must understand the importance of the content of counselling given before and during family members' bedside visits in the ICU. Moreover, ICPs should ensure that they adopt a holistic approach to the content of counselling, which should include information on both coping in the present and recovery in the future. Family members felt that ICPs did not take adequate care to verify that they understood the issues discussed during counselling and that they lacked opportunities to give feedback. Efforts should therefore be made to ensure that counselling is dialogue‐based rather than merely informative.

## FUNDING INFORMATION

This research was not supported by any specific grant from funding agencies in the public, commercial, or not‐for‐profit sectors.

## CONFLICT OF INTEREST STATEMENT

None.

## ETHICAL APPROVAL

The study was approved by the Research Ethics Committee of the health care district of Northern Ostrobothnia Hospital (EETTMK: 28/2016) and authorized by all five Finnish university hospitals. Participation in the study was voluntary and anonymous, and data were collected in compliance with the concept of privacy by design and default established in the general data protection regulation (GDPR) of the European Union. The researchers cannot identify which intensive care professionals did or did not respond to the survey. The collected electronic data do not include personal data and were saved on a computer secured with a password. Only the researchers had access to this computer.

## Data Availability

For legal reasons, the data used in this work cannot be transferred to a third‐party repository. However, it is available from the first author upon request.
